# Bioavailability of Oral Ondansetron in Dogs: A Crossover Study

**DOI:** 10.1111/jvp.70024

**Published:** 2025-09-09

**Authors:** Amanda Garrick, Kristin Zersen, Daniel Gustafson, Jessica Quimby, Amanda Diaz, Sarah Shropshire

**Affiliations:** ^1^ Clinical Sciences Department Colorado State University Fort Collins Colorado USA

## Abstract

The purpose of this study was to evaluate the pharmacokinetics of oral (PO) ondansetron compared to intravenous (IV) ondansetron in eight healthy client‐owned dogs. Dogs were randomized to one of two protocols in a crossover design, receiving PO or IV ondansetron at a dose of 1 mg/kg on Day 0 and the opposite formulation at an equal dose on Day 7. Plasma was collected at baseline and 1, 2, 4, and 8 h post administration. Ondansetron concentrations were measured utilizing liquid chromatography/mass spectrometry. For IV administration, AUC_0–8h_ was 1181 ± 619 ng/mL*h, with all dogs having detectable plasma concentrations at all time points. For PO administration, mean *C*_max_ was 22 ± 11.3 ng/mL and AUC_0–8h_ was 61.7 ± 45.4 ng/mL*h, with all dogs having undetectable concentrations at various time points. Oral mean bioavailability was estimated at 5.2% ± 2.1%. Oral bioavailability of ondansetron is very low in healthy dogs, raising concern for the efficacy of ondansetron when given orally at 1 mg/kg. Future studies evaluating pharmacodynamics of ondansetron in nauseous client‐owned dogs should be performed to investigate whether plasma drug concentrations are the optimal way to assess the efficacy of oral ondansetron.

## 1 Introduction

Ondansetron is a 5‐HT_3_ serotonin receptor antagonist that is commonly prescribed as an anti‐nausea and anti‐emetic medication for veterinary patients. Despite the clear demand for anti‐nausea therapies, there are limited anti‐nausea and anti‐emetic medications available for use in veterinary patients, which include maropitant, ondansetron, and to a lesser extent, metoclopramide (Elwood et al. 2010). While maropitant, an NK1 receptor antagonist, is a potent anti‐emetic, its anti‐nausea properties are considered inferior compared to injectable ondansetron (Kenward et al. 2017). Metoclopramide, a dopaminergic D2 antagonist, also has weak 5‐HT3 inhibitory effects, is a 5‐HT4 agonist, and is more commonly used clinically as a promotility agent (Elwood et al. 2010). Information on its anti‐nausea effect is variable, with some studies supporting its efficacy in certain populations, such as dogs with parvoviral enteritis (Yalcin and Keser 2017); however, other studies in the dog have shown it to have no appreciable effect on experimentally induced nausea from cisplatin (Kenward et al. 2017). Furthermore, the short half‐life of metoclopramide in the dog makes it impractical for intermittent oral dosing (Kenward et al. 2017). Given the paucity of data for a strong anti‐nausea effect of maropitant and metoclopramide, ondansetron is often utilized in clinical practice in an attempt to control nausea in hospital and, via the oral formulation, at home.

In humans, ondansetron is used routinely in hospital and outpatient settings and has been shown to have good absorption and efficacy via both oral and intravenous routes (Wilde and Markham 1996). Despite its relatively frequent clinical use, there is limited pharmacokinetic data comparing the efficacy of injectable versus oral administration in veterinary patients. Additionally, there is a lack of data for optimal oral dosing in dogs and cats.

Previous studies evaluating the pharmacokinetics of IV ondansetron found that it resulted in decreased nausea in dogs, displayed linear pharmacokinetics, and achieved serum concentrations within the previously published therapeutic range (Kenward et al. 2017) as extrapolated from previous canine studies for all dosing protocols tested (Sotelo et al. 2022; Kenward et al. 2017). Conversely, evaluation of PO administration of ondansetron has thus far found it to have suspected poor bioavailability and therefore suspected poor efficacy in dogs (Molli et al. 2022). This is consistent with prior data showing ondansetron to have relatively low oral bioavailability in multiple species due to high first‐pass metabolism in the gastrointestinal and hepatobiliary systems (Yang and Lee 2008; Saynor and Dixon 1989). The purpose of this study was to evaluate the pharmacokinetics of IV compared to PO ondansetron in a population of healthy client‐owned dogs.

## 2 Materials and Methods

### 2.1 Animals

This prospective, randomized, crossover study was approved by the Colorado State University Clinical Review Board, and all owners gave informed written consent to participate (Data S1). Eight clinically healthy client‐owned dogs were enrolled. A pre‐screening complete blood count and chemistry were offered at no charge to clients as an enrollment incentive. To be eligible for the study, dogs were required to be between the ages of 1 and 8 years, at least 5 kg, below a BCS of 8/9, and with no evidence of historical or active renal or liver disease based on bloodwork. Dogs were excluded if any anti‐emetic medications (metoclopramide, ondansetron, or maropitant) had been administered within 48 h of enrollment. To be considered for enrollment, dogs could not be on any medications aside from monthly preventatives. All dogs underwent a physical examination and had a complete blood count and serum chemistry panel performed at no charge to clients as an enrollment incentive and to rule out any concurrent underlying disease processes.

### 2.2 Materials

Oral ondansetron (ondansetron hydrochloride, 4 mg and 8 mg tablets; Rising Health LLC; Saddle Brook, NJ) was purchased through the pharmacy at Colorado State University Veterinary Teaching Hospital. Injectable ondansetron was purchased from West/Ward Pharmaceuticals (Eatontown, NJ, USA) through the Colorado State University Veterinary Teaching Hospital.

### 2.3 Ondansetron Dosing

Peripheral IV catheters were placed in all patients on both study days for ease of blood sampling and IV administration of ondansetron, when indicated. All dogs were fasted in the morning of each study day, and all dogs were dosed at the same time of day, which was in the morning. Dogs were randomized into one of two groups: 1 mg/kg ondansetron IV on Day 0 and 1 mg/kg ondansetron PO on Day 7 (Group AB) or 1 mg/kg ondansetron PO on Day 0 and 1 mg/kg ondansetron IV on Day 7 (Group BA). One dog in Group AB returned on Day 10 rather than Day 7 due to owner time conflicts. Oral ondansetron was administered in a soft treat, and all dogs were observed to eat this treat in its entirety.

### 2.4 Blood Sampling

Blood (1.3 mL) was collected prior to drug administration (baseline) and at 1, 2, 4, and 8 h after administration of ondansetron each day. After collection, blood was transferred into lithium heparin microtainers (JorVet, Loveland, CO) and refrigerated prior to centrifugation. Plasma was separated by centrifugation within 60 min of collection and frozen in aliquots at −80°C for batched analysis to assess plasma ondansetron concentrations at each of these timepoints.

### 2.5 Ondansetron Analysis

Plasma ondansetron concentrations were measured using a liquid chromatography coupled to tandem mass spectrometry (LCMS) assay based on a previously published method (Quimby et al. 2014), modified and previously published for analysis of canine samples (Sotelo et al. 2022). Standard and the quality control (QC) samples were prepared with the addition of 5 mL of known amounts of ondansetron that were dissolved in methanol:MilliQ water (1:1) and also with 5 mL of the internal standard (IS) zolpidem (50 ng/mL in a 1:1 methanol:MilliQ water to 50 mL of blank canine plasma) followed by mixing well. Unknown samples were then prepared with the addition of 5 mL of 1:1 methanol:MilliQ water and 5 mL of the IS mixture, followed by mixing well. The unknown samples, standard samples, and QC samples were then extracted by adding 500 mL of 1:1 pentane: ethyl acetate with 0.1% ammonium hydroxide. This was then mixed on a vortex shaker for 10 min. Samples were then centrifuged for 10 min at 13,300 × ***g***, followed by 400 mL of the organic phase (top) being transferred to a fresh microcentrifuge tube and evaporated to dryness on a rotary evaporator which was operated at medium heat. The samples were then reconstituted in 100 mL of 1:1 methanol:MilliQ water with 0.1% acetic acid and then transferred to auto‐sample vials with low volume glass inserts. Using an ABI 3200 QTrap triple quadrupole mass spectrometer with an Agilent 1200 LC system and HTC‐Leap autosampler, samples were analyzed. The sample volume that was injected was 10 μL and chromatography was performed using a Waters Phenyl 2.5 mm 4.6 × 50 mm LC column with a Phenomenex C18 filter frit guard cartridge. The mobile phase was made up of a gradient system using methanol and 10 mM ammonium acetate with 0.1% acetic acid with initial concentrations of 10% methanol that was increased linearly from 1.0 to 2.0 min to 98% methanol where it was then held until 3.5 min when the starting concentration of 10% methanol was re‐established linearly over 0.5 min and held for the remainder of the 5.0 min run. Multiple reaction monitoring under positive ion mode of ondansetron at 294.3 *m/z* à170.2 m/z and zolpidem at 308.3 *m/z* à235.2 *m/z* using unit resolution for Q1 and Q3, ion spray voltage of 5500 V, source temperature of 500°C, curtain gas of 35, ion source gases 1 and 2 at 60, and collision gas at medium was used for detection of ondansetron and the IS (zolpidem). Compound dependent parameters (DP, depolarizing potential; EX, exit potential; CEP, collision entrance potential; CE, collision energy; and CXP, collision exit potential) were then developed using instrument optimization algorithms. Assay performance based on QC sample analysis at 3.9, 15.6, 62.5, and 250 ng/mL showed an accuracy and precision (CV%) of 90.4% ± 3.3% with 16/20 (80%) QC samples showing an accuracy > 85%. Batch acceptance was based on > 75% of QC samples having an accuracy > 85% and the LLOQ for the analysis was 3.9 ng/mL based on the lowest concentration at least twofold above baseline and having an accuracy > 80%. Ondansetron concentrations below the LLOQ were set at 1 ng/mL for statistical analysis.

### 2.6 Pharmacokinetic and Statistical Analysis

Pharmacokinetic parameters between PO and IV routes were compared using area under the curve (AUC) over the 8‐hour collection period (AUC_0‐8_). AUC values were calculated using the linear trapezoid method with linear interpolation. For PO administration, the maximum plasma concentration (*C*_max_), the time to maximum plasma concentration (*T*_max_), and calculated bioavailability were also assessed.

Normality of data was assessed with a Shapiro–Wilk test. The AUC_0–8_ IV versus PO administration was compared using paired *t*‐tests. All statistical analysis was performed using Prism Software (Prism 9; GraphPad, La Jolla, CA, USA).

### 2.7 Sample Size Calculation

Preliminary data to determine appropriate power was not available, but similar crossover studies have used six to eight healthy animals, so a total of eight dogs was utilized (Giorgi et al. 2012; Jay et al. 2013; Chan et al. 2015).

## 3 Results

Eight healthy dogs were enrolled in the study. The ages of the dogs enrolled had a range of 5 years (2–7 years) with a median age of 4.5 years. Two males and 6 females were included; all dogs were neutered or spayed. The majority of the dogs (4) were mixed breed; the other 4 dogs included one Doberman, one Labrador, one Golden Retriever, and one Australian Shepherd. The smallest dog was 19.5 kg and the largest 35.5 kg; dogs had a mean weight of 26.4 kg. All dogs had unremarkable physical examinations and unremarkable CBCs and serum biochemistry panels prior to beginning the study. All 8 dogs enrolled completed the study and no adverse reactions were observed.

### 3.1 Ondansetron Concentration in Plasma

**FIGURE 1:**
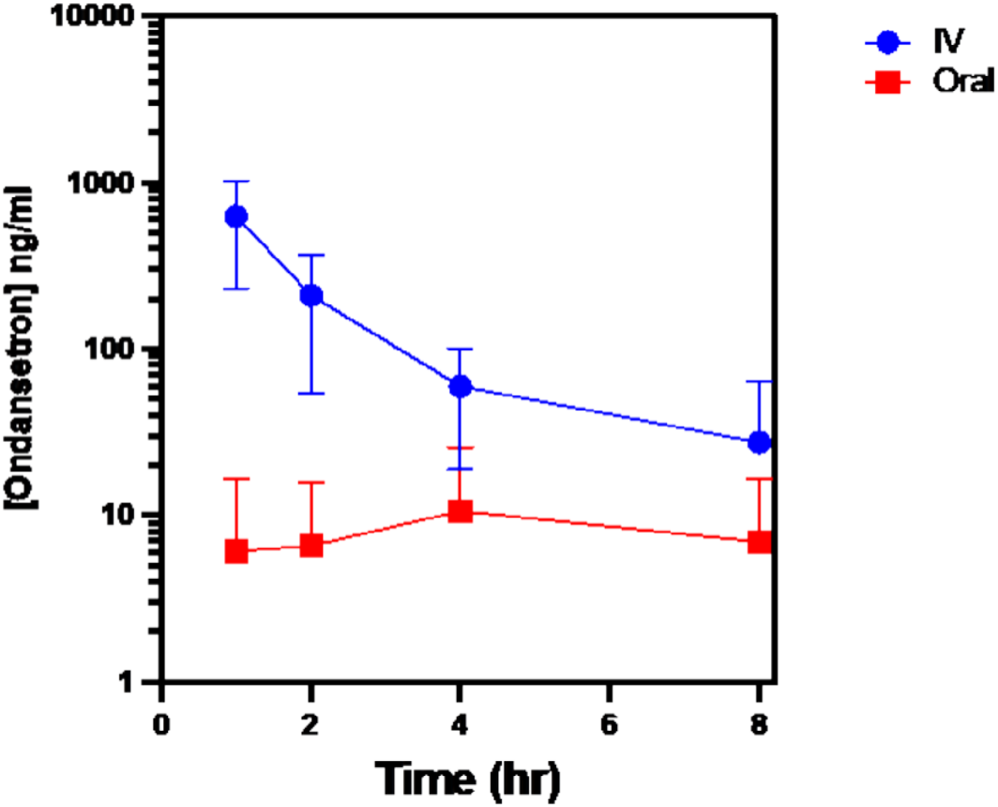
Ondansetron plasma concentration (mean ± standard deviation, ng/mL) versus time (hours) in 8 client‐owned healthy dogs.

The plasma concentration of ondansetron in plasma versus time for IV and PO administration is shown in Figure 1. Ondansetron administered via the IV route displayed linear pharmacokinetics, with all dogs having detectable plasma drug concentrations at all timepoints. Oral dosing *C*_max_ was 22 ± 11.3 ng/mL, and the time to maximum plasma concentration was variable. All dogs given oral ondansetron had undetectable plasma drug concentrations at multiple timepoints, with two dogs having detectable concentrations recorded at only the 8‐h timepoint.

### 3.2 Pharmacokinetic Parameters

Pharmacokinetic parameters for IV and oral dosing are shown in Table 1. The time to maximum concentration (*T*_max_) of the PO administration group was 4.6 h, although the *T*_max_ plasma concentration varied considerably, with at least one dog reaching *C*_max_ at every recorded time point. Oral bioavailability was low (5.2%).

**TABLE 1:** *C*_max_, AUC, *T*_max_, and bioavailability (*F*) of ondansetron in 8 client‐owned dogs administered 1 mg/kg of ondansetron orally or IV.

PK parameter	IV	Oral
*C*_max_ (ng/mL)	—	22.0 ± 11.3 (7.0–43.7)
AUC_0–8h_ (ng/mL × h)	1181 ± 619 (499–1954)	61.7 ± 45.4 (14.0–151)
*T*_max_ (h)	—	4.6 ± 3.0 (1.0–8.0)
*F* (%) (estimated)	—	5.2 ± 2.1 (1.7–7.9)

*Note:* ± SD with the range in parentheses below.

**FIGURE 2:**
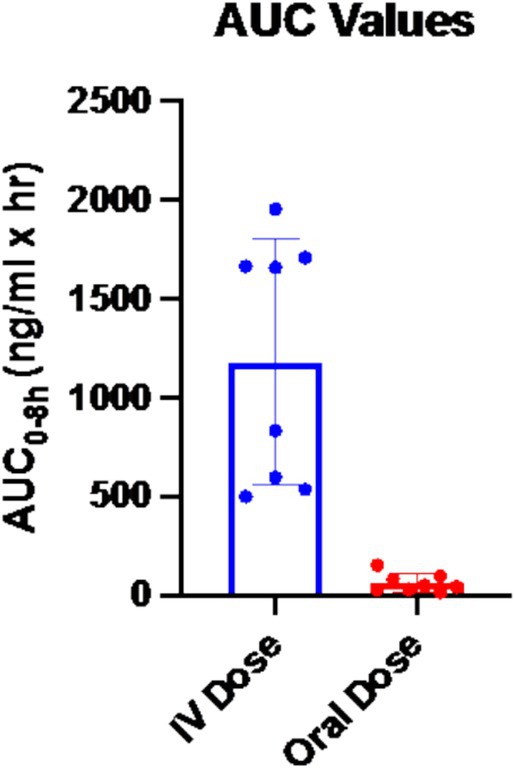
AUC_0–8_ values (ng/mL × h) for IV and PO dosing of ondansetron. The AUC of the drug when given IV was significantly higher (*p* = 0.0002) compared to oral administration.

AUC values are shown in Table 1 and graphically in Figure 2. Mean AUC_0–8h_ was 1181 ± 619 ng/mL*h for IV administration. Mean AUC_0–8h_ was significantly lower at 61.7 ± 45.4 ng/mL*h for PO administration (*p* < 0.0001).

## 4 Discussion

This study investigated the pharmacokinetics, including bioavailability, of oral and intravenous ondansetron in a crossover model. Oral ondansetron has been shown to have low bioavailability in the dog previously (Saynor and Dixon 1989; Molli et al. 2022); however, the crossover design employed here was intended to account for variation in metabolism among individuals. The dogs enrolled in this study were also client‐owned healthy individuals rather than a more homogenous purpose‐bred population, as has been utilized in some other publications, possibly resulting in better representation of a diverse patient population.

The oral bioavailability of ondansetron is very low in healthy dogs, raising concern for the efficacy of this drug when given orally at 1 mg/kg, consistent with previous publications (Saynor and Dixon 1989; Molli et al. 2022). Despite this, there are anecdotal accounts of oral ondansetron resulting in observable anti‐nausea effects. In most studies investigating the anti‐nausea effects of ondansetron, the medication was given intravenously (Yalcin and Keser 2017; Sotelo et al. 2022; Kenward et al. 2017). However, at least one study described an improvement in nausea in dogs given oral ondansetron at 0.5 mg/kg compared to controls, although this effect was inferior to PO maropitant (Burke et al. 2022). Plasma concentrations of the drug were not incorporated into this study, but further studies pairing plasma concentrations with clinical nausea scores of sick animals after giving oral ondansetron could help to determine if plasma concentrations of this drug correlate with clinical effect.

It is also possible that ondansetron administered orally in the dog is having effects not adequately reflected by the concentrations achieved in plasma. While ondansetron in veterinary species is thought to primarily target the centrally located 5‐HT3 receptors in the chemoreceptor trigger zone, there are numerous serotonin receptors present in the gut; in humans, the majority of 5‐HT3 receptors in the body are located in enterochromaffin cells in the gastrointestinal mucosa (Mawe and Hoffman 2013). Studies in dogs and rats have shown that ondansetron can reduce or prevent fat‐associated ileus, thus improving peristalsis and GI transit time (Lin and Chen 2003), and high levels of serotonin and serotonin signaling molecules in the gut lumen have been associated with irritable bowel syndrome in humans, particularly in cases presenting with poor GI motility (Spiller 2011; Vahora et al. 2020), suggesting that blockade of serotonin receptors at the local level may have benefits beyond its central anti‐nausea effect.

It is also worth considering that the pharmacokinetics of orally dosed ondansetron may differ in dogs with significant renal or hepatic dysfunction. In humans with hepatic insufficiency, first‐pass metabolism of ondansetron has been shown to be significantly reduced and AUC increased (Figg et al. 1996). Renal insufficiency has not been associated with altered ondansetron pharmacokinetics in humans; however, one feline study does suggest decreased clearance and increased AUC in cats with renal disease, although to a lesser extent compared to those with hepatic dysfunction (Fitzpatrick et al. 2016). As many veterinary patients with severe renal or hepatic disease present clinically with nausea, this is a commonly prescribed drug in these populations and could result in higher plasma concentrations in these groups.

Limitations of our study included a relatively small sample size and an uneven number of males and females. All dogs in the study were spayed or neutered, which likely minimized the impact of this difference. There was a single dog in group AB who received the oral dose 10 days after the IV dose rather than 7 days. Based on the pharmacokinetic action of this drug and the fact that all dogs had a baseline plasma concentration of 0 on Day 7, this was not expected to impact results. An additional limitation that should be considered is that the blood sampling regimen could have been optimized to sample for a longer period of time (> 24 h) to further assess the bioavailability of ondansetron in this population of dogs.

In conclusion, the oral bioavailability of ondansetron in healthy dogs is extremely low, raising concern for the efficacy of this drug when given orally at 1 mg/kg. Future studies evaluating the pharmacodynamics of ondansetron in nauseous client‐owned dogs should be performed to investigate whether plasma drug concentrations are the optimal way to assess the efficacy of oral ondansetron. Studies investigating plasma concentrations and anti‐nausea effects specifically in populations affected by renal and hepatic disease could also shed light on populations where the use of oral ondansetron may be more effective.

## Author Contributions

A.G.: study design, data collection, preparation of manuscript. K.Z.: study design, data collection, resources, revised the manuscript. D.G.: data analysis. J.Q.: study design, validation, revised the manuscript. A.D.: study design. S.S.: study design, data collection, resources, revised the manuscript. All authors have read and approved the final manuscript.

## Ethics Statement

The authors declare that this study was approved by and conducted in accordance with the requirements of the University of Colorado Institutional Animal Care and Use Committee (Protocol #4493). The authors confirm that they have adhered to US standards for the protection of animals used for scientific purposes.

## Conflicts of Interest

The authors declare no conflicts of interest.

## Supporting information


**Data S1:** jvp70024‐sup‐0001‐supinfo.pdf.

## Data Availability

The data that support the findings of this study are available from the corresponding author upon reasonable request.
