# Supplementary material for: Bioavailability of Oral Ondansetron in Dogs: A Crossover Study
Source: J Vet Pharmacol Ther. 2025 Sep 9;49(1):17–21. doi: 10.1111/jvp.70024 (PMC12796776; doi:10.1111/jvp.70024)
Supplement: Supplementary file 1 — Data S1: jvp70024‐sup‐0001‐supinfo.pdf. [file JVP-49-17-s001.pdf]

Colorado State University
JAMES L. VOSS
VETERINARY TEACHING HOSPITAL

Place Patient Identification Sticker Here

Appointment Desk: (970) 297-5000
http://csu-cvmbs.colostate.edu/vth/

Clinical Trials: Owner Informed Consent

The Pharmacokinetics of Oral and Intravenous Ondansetron: A Crossover Study

Ondansetron is commonly prescribed to treat vomiting in dogs and cats, however, we don't know how the drug is absorbed or distributed throughout the body. This information is important to ensure we are dosing the medication appropriately. This study will measure the amount of ondansetron in the blood after giving the medication, which will allow us to determine this important information.

I understand that the veterinarians at this hospital are doing research to improve animal health, animal care, and education. The details of '*The Pharmacokinetics of Oral and Intravenous Ondansetron: A Crossover Study'* Clinical Trial have been explained to me by:

Dr. ___________________________ on __________________________.

Please initial the following lines to note your understanding of the parts of this study:

_______ My dog must be healthy and should not be taking any medications to be a part of this study.

_______ The purpose of this study is to measure ondansetron drug levels in the blood so pharmacokinetic parameters can be assessed.

_______ As part of this study, my dog will need to be in the hospital for 10 hours per day for a total of 2 days. The two study dates will be one week apart.

_______ As part of this study, my dog will have a single dose of oral ondansetron given on one study day and a single dose of IV ondansetron given on the second study day. On each study day, an IV catheter will be placed and blood samples will be collected from the IV catheter. If necessary, we may need to draw blood directly from a vein. My dog will have a total of 1.5 teaspoons of blood collected on each study day, which is a safe amount given my dog's weight.

_______ I realize there is no direct benefit to my dog from being a part of this study.

_______ All drugs and steps used have been carefully tested to decrease the chance of negative side effects. However, I realize it is possible my dog will have unexpected side-effects which could be mild, moderate, or severe (including death). The chance of having an allergic reaction to ondansetron is low, but is possible. IV catheter placement and blood draws may result in the formation of a hematoma (blood blister) or inflammation at the site. My dog will be watched closely for side effects and corrective action will be taken if needed.

_______ I understand that I will not incur any costs associated with this study. My dog will have a CBC and chemistry panel, and urinalysis performed at no charge to me and the results will be provided to me.

_______ I understand that the costs associated with adverse events are my responsibility.

_______ I understand that I must return to CSU for both study dates.

_______ I understand that information and samples collected during this study are the property of the investigator and may be stored for future use.

_______ I give my permission to publish information and photos obtained from this study for the benefit of the scientific community. I understand that my pet will not be identified individually.

_______ I may remove my dog from this study without penalty.

_______ The veterinarian in charge may remove my dog from this study if he/she determines that it is not benefiting or is harming my dog

_______ I may discuss this study with my own veterinarian and ask his/her advice.

_______ I understand that someone may contact me after my dog has finished this study to collect follow-up information. This may occur several months to years following the end of the trial.

_______ I have had time to ask questions about this study and feel comfortable enrolling my dog in this study based on the information provided.

_______ I understand that the funding for this study is provided by a Young Investigator's Grant from the Center for Companion Animal Studies.

_______ The investigators in this study declare no conflicts of interest.

As a result of discussion with Dr. ________________________, and after reading the above, I voluntarily consent to participate in this project and will follow the instructions of the veterinarians-in-charge as it pertains to therapy and follow-up tests.

Signed ____ Date _____________________

Owner or authorized agent of the owner

Witnessed By: ____ Date _____________________

For questions about this study, please contact: *Kristin Zersen (Kristin.zersen @colostate.edu) or Sarah Shropshire (Sarah.shropshire@ colostate.edu).*

For questions about the ethical conduct of animal research at Colorado State University, please go to Reporting Animal Welfare Concerns website: *https://www.research.colostate.edu/ricro/iacuc/reporting-animal-welfare-concerns/ or email IACUC Staff in RICRO: RICRO_IACUC@mail.colostate.edu*
